# Supplementary material for: Beyond 2D cell cultures: how 3D models are changing the in vitro study of ovarian cancer and how to make the most of them
Source: PeerJ. 2024 Aug 29;12:e17603. doi: 10.7717/peerj.17603 (PMC11366228; doi:10.7717/peerj.17603)
Supplement: Supplemental Information 1 [file peerj-12-17603-s001.zip › Dichiarazione integrativa DGUE fino 40.0000_EN - no PNRR.docx]

**SUPPLEMENTARY DECLARATION TO THE ESPD**

I, the undersigned …………………………………………………………………………………………………………………………………………………………

born in …………………………………………………. on …………………………….………… Tax ID no. ………………………………………………….

in my capacity as ………………………………………………….………………………………………………….………………………………………………….

of the economic operator ………………………………………………………………………………………………………………………………………………

If the bid is submitted by a representative with special power of attorney, please indicate the details of the notarial instrument ………………………………………

……………………………………………………………………………………………………………………………………………………………………………………….

with registered office in …………………………………………………………………… Certified email (PEC) ………………………………………………………………………………………

Tax identification/VAT number of the economic operator …………………………………………………………….………………………………………

Business activity code of the company: ……………………………….……………………………….………………………, for the purposes of participating in the above-said procedure, under my own responsibility pursuant to Article 38, paragraph 3 and Articles 46, 47 and 77-bis of Presidential Decree 445/2000, as amended, aware of the criminal penalties for misrepresentation and forgery under Article 76 of Presidential Decree 445/2000, which apply in addition to exclusion from the tender procedure,

**Declare as follows** *(Tick the appropriate boxes)*:

- I am aware of the obligations arising from the commissioning body’s Code of Conduct, adopted by Rector’s Decree no. 1408/14 of 01/10/2014 and available via the link <https://www.unibo.it/it/ateneo/bandi-di-gara/obblighi-di-comportamento>. In the event of being awarded the contract, I undertake to comply and ensure employees and collaborators’ compliance, where applicable, with the aforementioned Code, failing which the contract may be terminated;
- *[for services/supplies in the sensitive sectors referred to in Article 1, paragraph 53 of Law 190/2012]* I am registered on the White List of suppliers and service providers not subject to mafia infiltration attempts, kept at the Prefecture of the Province of ________________, or I have applied to register on the White List of suppliers and service providers not subject to mafia infiltration attempts, kept at the Prefecture of the Province of ________________;

| **For economic operators which are not resident and have no permanent establishment in Italy**   - I undertake, in the event of being awarded the contract, to comply with the provisions of Article 17, paragraph 2 and Article 53, paragraph 3 of Presidential Decree 633/1972 and to communicate the tax representative’s name to the commissioning body, as required by law; - I provide the following information: tax domicile, Tax ID number, VAT number, certified email address _________________________________________________ **or** equivalent tool in another Member State, for the purpose of receiving notices under Article 90 of the Code; |
| --- |

- I have read and accept the processing of personal data as set out in the [Privacy policy applicable to businesses and goods and service providers – University of Bologna (unibo.it)](https://www.unibo.it/it/ateneo/privacy-e-note-legali/privacy/informativa-per-operatori-economici-e-fornitori-di-lavori-beni-e-servizi);
- *[To be added when the supply/service requires appointment of a processor for third-party data. To be removed if the service/supply only provides for the processing of the personal data of the contracting parties]* I meet the requirements of experience, capacity and reliability needed to ensure full compliance with the provisions on personal data protection, including with regard to security, and I am in a position to assume the role of Processor. I am aware that, in the event of being awarded the contract, I will be appointed by the Administration as a “Processor” or “Sub-Processor” for the processing of personal data, pursuant to Article 28 of Regulation (EU) 2016/679 of the European Parliament and of the Council of 27 April 2016, laying down the General Data Protection Regulation (hereinafter also referred to as the “EU Regulation” or “GDPR”), as well as the Italian provisions implementing the GDPR, subject to verification by the Administration that the national and EU legal requirements are met. In that case, I undertake to provide the Administration with sufficient guarantees and implement all technical and organisational measures necessary to ensure compliance with the laws and standards in force on the processing of personal data, and any further measures needed to comply with any change in said laws such to result in new requirements (including of a physical, logical, technical or organisational nature, relating to security or processing of personal data) for the Processor or Sub-Processor, and to collaborate, within the limits of my technical and organisational responsibilities and resources, with the Controller/Processor in order to develop and adopt corrective measures to comply with any new requirements and measures implemented during performance of the contract, at no additional cost for the Administration;

| **For economic operators established, resident or domiciled in so-called “blacklisted” countries**   - I have a valid authorisation issued in accordance with Ministerial Decree dated 14 December 2010 of the Ministry of the Economy and Finance (pursuant to Article 37 of Decree-Law 78/2010, converted into Law 122/2010) **or** I have submitted a request for authorisation pursuant to Article 1, paragraph 3 of Ministerial Decree dated 14.12.2010 and I attach a true copy of the application submitted to the Ministry; |
| --- |

| **For economic operators in an arrangement with creditors on a going concern basis pursuant to Article 186-bis of Royal Decree no. 267 dated 16 March 1942**   - Furthermore, pursuant to Articles 46 and 47 of Presidential Decree 445/2000, the bidder provides details concerning the arrangement with creditors and the authorisation to participate in tenders granted to it. The bidder will not act in the tender as the leader of a temporary association of companies; no other company in the temporary association is subject to any insolvency proceedings pursuant to Article 186-bis, paragraph 6 of Royal Decree no. 267 dated 16 March 1942.   The bidder submits a report drafted by a qualified professional pursuant to [Article 67](http://bd01.leggiditalia.it/cgi-bin/FulShow?TIPO=5&NOTXT=1&KEY=01LX0000107749ART67), third paragraph, letter d) of Royal Decree no. 267 dated 16 March 1942, certifying its compliance with the plan and reasonable ability to comply with the contract. |
| --- |

- Participation in this procedure will cause no conflict of interest or potential conflict of interest under Articles 6 and 7 of Presidential Decree 62/2013 and Article 16 of the Code and I undertake to timely report any potential conflict of interest affecting anyone involved in the procedure and submit any useful information for consideration to the commissioning body;
- Pursuant to Article 53, paragraph 16-ter of Legislative Decree 165/2001 (prohibition of *pantouflage* or revolving door), I have not concluded employment or freelance contracts with and, in any case, have not engaged former employees of the commissioning body whose employment with the latter terminated less than three years ago and who, during the last three years of service, exercised authorisation or negotiation powers on behalf of the commissioning body vis-a-vis the same economic operator. I am aware that any contracts or engagements in breach of the prohibition pursuant to Article 53, paragraph 16-ter of Legislative Decree 165/2001 are null and void and that the private entities which entered into such contracts or engagements may not contract with the public administration for the following three years and will be required to return any consideration received in relation thereto;

Signature

[Digitally signed by the legal representative/attorney^[[1]](#footnote-1)^]

1. If the declaration is signed by an attorney of the legal representative, please attach a true copy of the power of attorney or, only in the event that the Economic Operator’s Chamber of Commerce certificate expressly indicates the representation powers granted to the attorney, a self-declaration of the attorney/legal representative who signs this document, certifying the representation powers stated in the Chamber of Commerce certificate. [↑](#footnote-ref-1)
