# Supplementary material for: Beyond 2D cell cultures: how 3D models are changing the in vitro study of ovarian cancer and how to make the most of them
Source: PeerJ. 2024 Aug 29;12:e17603. doi: 10.7717/peerj.17603 (PMC11366228; doi:10.7717/peerj.17603)
Supplement: Supplemental Information 1 [file peerj-12-17603-s001.zip › Dichiarazione integrativa DGUE fino 40.0000_EN.docx]

**SUPPLEMENTARY DECLARATION TO THE ESPD**

I, the undersigned …………………………………………………………………………………………………………………………………………………………

born in …………………………………………………. on …………………………….………… Tax ID no. ………………………………………………….

in my capacity as ………………………………………………….………………………………………………….………………………………………………….

of the economic operator ………………………………………………………………………………………………………………………………………………

If the bid is submitted by a representative with special power of attorney, please indicate the details of the notarial instrument ………………………………………

……………………………………………………………………………………………………………………………………………………………………………………….

with registered office in …………………………………………………………………… Certified email (PEC) ………………………………………………………………………………………

Tax identification/VAT number of the economic operator …………………………………………………………….………………………………………

Business activity code of the company: ……………………………….……………………………….………………………, for the purposes of participating in the above-said procedure, under my own responsibility pursuant to Article 38, paragraph 3 and Articles 46, 47 and 77-bis of Presidential Decree 445/2000, as amended, aware of the criminal penalties for misrepresentation and forgery under Article 76 of Presidential Decree 445/2000, which apply in addition to exclusion from the tender procedure,

**Declare as follows** *(Tick the appropriate boxes)*:

***[For NRRP procurement]* Pursuant to anti-money laundering law** (Legislative Decree 231/2007, particularly Article 2 of Technical Annex and Legislative Decree 125/2019)

- I am the sole beneficial owner of the economic operator;
- The company has no beneficial owner, since ………………………………………………… [please specify];
- I am the beneficial owner together with: [please provide the personal information of any beneficial owner(s) other than the declarant. Repeat the information concerning beneficial owners as many times as needed.]
- Name ……………………………. Surname ………………………… born in …… ………… on ……… - Tax ID no. ………………………………… ;
- Name ……………………………. Surname ………………………… born in ……………… on ……… - Tax ID no. …………………………………;

**(Each beneficial owner must attach a declaration of no conflict of interest)**.

| **HOW TO IDENTIFY THE BENEFICIAL OWNER**  In order to identify the beneficial owner, reference should be made to Legislative Decree 231/2007 (Article 2 of Technical Annex) and Legislative Decree 125/2019.  As a rule, there are 3 alternative methods to identify the beneficial owner:   1. **Ownership**: The person(s) who has/have an ownership interest of 25% or more in the company is/are identified as the beneficial owner(s). When this ownership interest is controlled by a third-party legal (i.e. non-natural) person, the complete ownership chain should be examined to identify the ultimate beneficial owner; 2. **Control**: The person or group of persons who exercise the most influence of all shareholders, through a majority of voting rights or contracts, are identified as the beneficial owner(s). This method is especially relevant when the beneficial owner cannot be identified based on ownership (see point 1); 3. **Residual method**: If no beneficial owner could be identified using the other two methods, the beneficial owner according to this method is the person who manages or directs the company. |
| --- |

- I unreservedly accept all the rules and provisions set out in the documentation ***[optional, if a CAM decree is in force]*** including the minimum environmental criteria according to decree … ***[reference of the decree is indicated by the commissioning body]***;

***[If applicable, for NRRP procurement] For tender procedures involving public investments funded, in whole or in part, by the National Recovery and Resilience Plan, pursuant to Regulation (EU) 2021/240 of the European Parliament and of the Council of 10 February 2021 and Regulation (EU) 2021/241 of the European Parliament and of the Council of 12 February 2021 (NRRP), or by the National Plan for Complementary Investments to the NRRP, pursuant to Article 1 of Decree-Law no. 59 dated 6 May 2021 (PNC), and initiated after entry into force of Decree-Law no. 77 dated 31 May 2021, converted with amendments by Law no. 108 dated 29 July 2021*:**

- The company has more than 50 employees. For this reason, I attach:
- A copy of the latest gender report on male and female staff submitted to the trade union representatives and regional equality councillors, and a certification of its conformity with that submitted to the trade union representatives and regional equality councillors pursuant to Article 47, paragraph 2 of Decree-Law 77/2021, converted into Law 108/2021 (Article 94, paragraph 5, letter c) of the Code);
- Additionally, if the report was not submitted by the deadline indicated in Article 46 of Legislative Decree 198/2006, proof of submission of the same report to the trade union representatives and regional equality councillors;

**or, alternatively**

- The company has 15 to 50 employees. For this reason,
- It undertakes, in the event of being awarded the contract, within 6 months of the signing, to submit a gender report on male and female staff, providing details for each job and the status of hiring, training, professional development, salary levels, career advancements, other mobility aspects, use of the wage supplementation scheme (*Cassa Integrazione Guadagni*), dismissals, early retirements and retirements, and salaries actually paid, and to send it to the trade union representatives and regional equality councillors (Article 47, paragraph 3-bis of Decree-Law 77/2021, converted into Law 108/2021);
- In the twelve months prior to bidding in this procedure, it has not breached the obligation to submit the gender report on male and female staff pursuant to Article 47, paragraph 3 of Decree-Law 77/2021, in relation to other public procedures under the NRRP or the PNC;
- It undertakes, in the event of being awarded the contract, within 6 months of the signing, to submit a declaration of compliance with the rules that govern the right of persons with disabilities to work, pursuant to Article 17 of Law no. 68 dated 12 March 1999, and a report regarding compliance with the obligations under the same Law 68/1999, as well as any sanctions or measures taken against it in the three-year period before the deadline to submit bids. By the same deadline, the report must be submitted to trade union representatives as well (Article 47, paragraph 3-bis of Decree-Law 77/2021, converted into Law 108/2021);

**or, alternatively**

- The company has fewer than 15 employees. For this reason, it is under no obligation to comply with Article 47, paragraphs 2, 3 and 3-bis of Decree-Law 77/2021, converted into Law 108/2021;
- The number of employees at the time of submitting the application is ___. Pursuant to Article 47, paragraph 4 of Decree-Law 77/2021, in the event of being awarded the contract and if three or more new employees were to be hired to perform the contract itself or any related or ancillary activities, the company undertakes to reserve:
- 30% of new hires for youth employment; *[Please indicate any different percentage, if the exception under Article 47, paragraph 7 of Decree-Law 77/2021 applies]*
- 30% of new hires for female employment; *[Please indicate any different percentage, if the exception under Article 47, paragraph 7 of Decree-Law 77/2021 applies]*

**Further declare as follows**

- I am aware of the obligations arising from the commissioning body’s Code of Conduct, adopted by Rector’s Decree no. 1408/14 of 01/10/2014 and available via the link <https://www.unibo.it/it/ateneo/bandi-di-gara/obblighi-di-comportamento>. In the event of being awarded the contract, I undertake to comply and ensure employees and collaborators’ compliance, where applicable, with the aforementioned Code, failing which the contract may be terminated;
- *[for services/supplies in the sensitive sectors referred to in Article 1, paragraph 53 of Law 190/2012]* I am registered on the White List of suppliers and service providers not subject to mafia infiltration attempts, kept at the Prefecture of the Province of ________________, or I have applied to register on the White List of suppliers and service providers not subject to mafia infiltration attempts, kept at the Prefecture of the Province of ________________;

| **For economic operators which are not resident and have no permanent establishment in Italy**   - I undertake, in the event of being awarded the contract, to comply with the provisions of Article 17, paragraph 2 and Article 53, paragraph 3 of Presidential Decree 633/1972 and to communicate the tax representative’s name to the commissioning body, as required by law; - I provide the following information: tax domicile, Tax ID number, VAT number, certified email address _________________________________________________ **or** equivalent tool in another Member State, for the purpose of receiving notices under Article 90 of the Code; |
| --- |

- I have read and accept the processing of personal data as set out in the [Privacy policy applicable to businesses and goods and service providers – University of Bologna (unibo.it)](https://www.unibo.it/it/ateneo/privacy-e-note-legali/privacy/informativa-per-operatori-economici-e-fornitori-di-lavori-beni-e-servizi);
- *[To be added when the supply/service requires appointment of a processor for third-party data. To be removed if the service/supply only provides for the processing of the personal data of the contracting parties]* I meet the requirements of experience, capacity and reliability needed to ensure full compliance with the provisions on personal data protection, including with regard to security, and I am in a position to assume the role of Processor. I am aware that, in the event of being awarded the contract, I will be appointed by the Administration as a “Processor” or “Sub-Processor” for the processing of personal data, pursuant to Article 28 of Regulation (EU) 2016/679 of the European Parliament and of the Council of 27 April 2016, laying down the General Data Protection Regulation (hereinafter also referred to as the “EU Regulation” or “GDPR”), as well as the Italian provisions implementing the GDPR, subject to verification by the Administration that the national and EU legal requirements are met. In that case, I undertake to provide the Administration with sufficient guarantees and implement all technical and organisational measures necessary to ensure compliance with the laws and standards in force on the processing of personal data, and any further measures needed to comply with any change in said laws such to result in new requirements (including of a physical, logical, technical or organisational nature, relating to security or processing of personal data) for the Processor or Sub-Processor, and to collaborate, within the limits of my technical and organisational responsibilities and resources, with the Controller/Processor in order to develop and adopt corrective measures to comply with any new requirements and measures implemented during performance of the contract, at no additional cost for the Administration;

| **For economic operators established, resident or domiciled in so-called “blacklisted” countries**   - I have a valid authorisation issued in accordance with Ministerial Decree dated 14 December 2010 of the Ministry of the Economy and Finance (pursuant to Article 37 of Decree-Law 78/2010, converted into Law 122/2010) **or** I have submitted a request for authorisation pursuant to Article 1, paragraph 3 of Ministerial Decree dated 14.12.2010 and I attach a true copy of the application submitted to the Ministry; |
| --- |

| **For economic operators in an arrangement with creditors on a going concern basis pursuant to Article 186-bis of Royal Decree no. 267 dated 16 March 1942**   - Furthermore, pursuant to Articles 46 and 47 of Presidential Decree 445/2000, the bidder provides details concerning the arrangement with creditors and the authorisation to participate in tenders granted to it. The bidder will not act in the tender as the leader of a temporary association of companies; no other company in the temporary association is subject to any insolvency proceedings pursuant to Article 186-bis, paragraph 6 of Royal Decree no. 267 dated 16 March 1942.   The bidder submits a report drafted by a qualified professional pursuant to [Article 67](http://bd01.leggiditalia.it/cgi-bin/FulShow?TIPO=5&NOTXT=1&KEY=01LX0000107749ART67), third paragraph, letter d) of Royal Decree no. 267 dated 16 March 1942, certifying its compliance with the plan and reasonable ability to comply with the contract. |
| --- |

- Participation in this procedure will cause no conflict of interest or potential conflict of interest under Articles 6 and 7 of Presidential Decree 62/2013 and Article 16 of the Code and I undertake to timely report any potential conflict of interest affecting anyone involved in the procedure and submit any useful information for consideration to the commissioning body;
- Pursuant to Article 53, paragraph 16-ter of Legislative Decree 165/2001 (prohibition of *pantouflage* or revolving door), I have not concluded employment or freelance contracts with and, in any case, have not engaged former employees of the commissioning body whose employment with the latter terminated less than three years ago and who, during the last three years of service, exercised authorisation or negotiation powers on behalf of the commissioning body vis-a-vis the same economic operator. I am aware that any contracts or engagements in breach of the prohibition pursuant to Article 53, paragraph 16-ter of Legislative Decree 165/2001 are null and void and that the private entities which entered into such contracts or engagements may not contract with the public administration for the following three years and will be required to return any consideration received in relation thereto;
- *[For NRRP procurement]* I undertake to comply with the “Do Not Significant Harm” (DNSH) principle, pursuant to Article 17 of Regulation (EU) 2020/852, Article 18 of Regulation (EU) 2021/241 and MEF-RGS Memo no. 33 dated 13.10.2022, “Do Not Significant Harm (so-called DNSH) Operational Guide Updates” in accordance with the Technical specifications and/or annexes.

Signature

[Digitally signed by the legal representative/attorney^[[1]](#footnote-1)^]

1. If the declaration is signed by an attorney of the legal representative, please attach a true copy of the power of attorney or, only in the event that the Economic Operator’s Chamber of Commerce certificate expressly indicates the representation powers granted to the attorney, a self-declaration of the attorney/legal representative who signs this document, certifying the representation powers stated in the Chamber of Commerce certificate. [↑](#footnote-ref-1)
