# Supplementary material for: Beyond 2D cell cultures: how 3D models are changing the in vitro study of ovarian cancer and how to make the most of them
Source: PeerJ. 2024 Aug 29;12:e17603. doi: 10.7717/peerj.17603 (PMC11366228; doi:10.7717/peerj.17603)
Supplement: Supplemental Information 1 [file peerj-12-17603-s001.zip › modulo tracciablità flussi_EN.doc]

**TRACEABILITY OF FINANCIAL FLOWS**

**PURSUANT TO LAW 136/2010**

**To:**

**Alma Mater Studiorum–Università di Bologna**

**____________**

**Certified email address: __________**

I, the undersigned _________________________________________________________ born on ____/____/______

Resident in ________________________________ Address __________________________________________________ Tax ID no. ___________________________________

as

⁭ Legal Representative

⁭ Representative with Power of Attorney, pursuant to _____________________________________________________

of the Economic Operator __________________________________________________________________________

with registered office in __________________________________________ Address ________________________________________ Tel. _____________________ Fax _____________________ Email _______________________________________ Tax ID/VAT no. ____________________________________________________________________

concerning *[scope of service/supply]*, **aware of the civil and criminal liability and penalties for misrepresentation pursuant to Article 76 of Presidential Decree 445/2000,**

**DECLARE**

- Pursuant to Article 3, paragraph 7 of Law no. 136 dated 13 August 2010, the details of the bank/postal current account into which the contract fee is to be paid are as follows: IBAN (Country, CIN EUR, CIN, ABI, CAB, account no.)

- The following persons are authorised to use the above-mentioned current account:

- Mr/Ms _____________________________ born in _______________________ on ____/____/______ Tax ID no. ______________________________ Resident in _________________________________ Address ___________________________________________________
- Mr/Ms _____________________________ born in _______________________ on ____/____/______ Tax ID no. ______________________________ Resident in _________________________________ Address ___________________________________________________

- Pursuant to Article 3 of Law no. 136 dated 13 August 2010, under penalty of the contract being rendered null and void, I undertake to comply with the laws on traceability of financial flows.

Place and date ________________

The undersigned

(Digitally signed)

_________________________
